# Supplementary material for: The Proinflammatory Role of ANGPTL8 R59W Variant in Modulating Inflammation through NF-κB Signaling Pathway under TNFα Stimulation
Source: Cells. 2023 Nov 2;12(21):2563. doi: 10.3390/cells12212563 (PMC10648545; doi:10.3390/cells12212563)

Supplementary-original gels

Figure2B

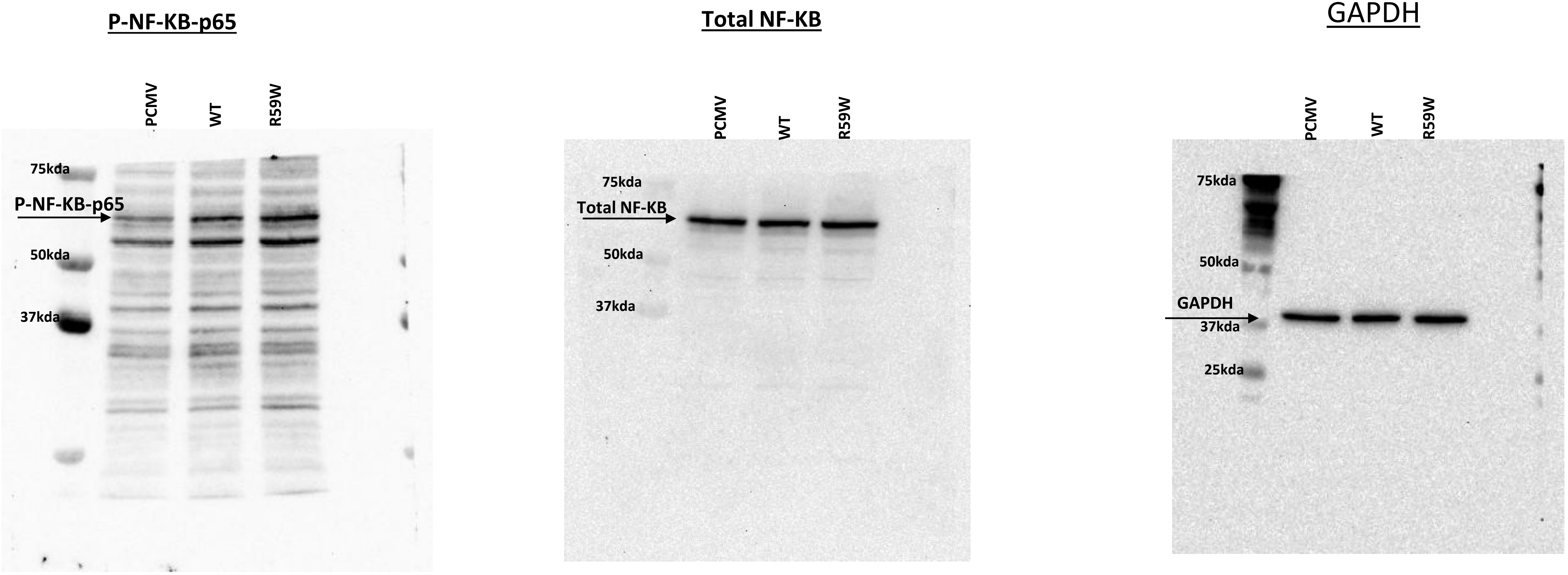

The blot above 75kda was cut and removed, the bottom half(below 75kda) was used for screening P-nf-KB

FIGURE-2C

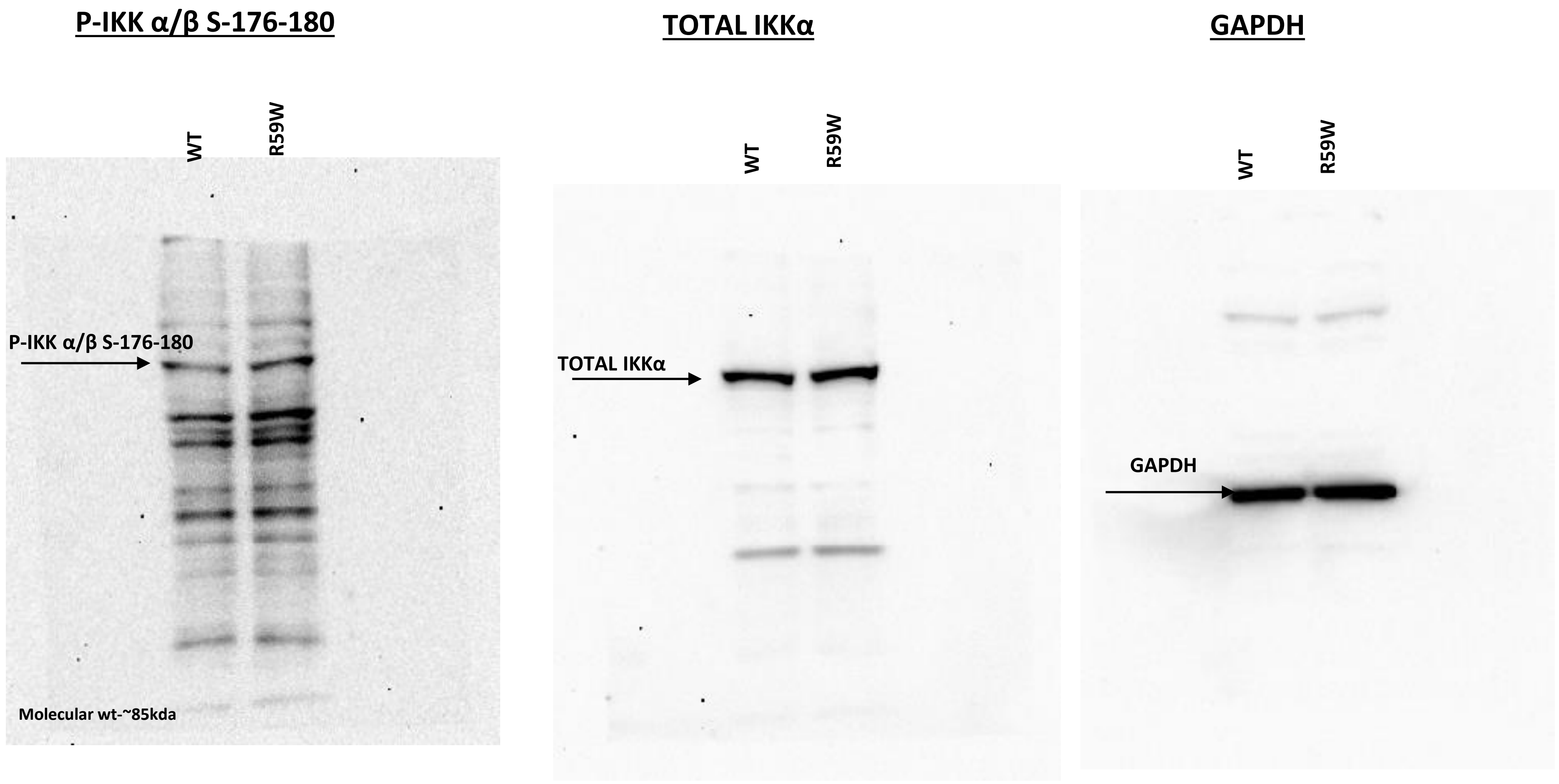
P-IKK α/β S-176-180
WT
R59W
P-IKK α/β S-176-180
Molecular wt-~85kda
TOTAL IKKα
WT
R59W
TOTAL IKKα
GAPDH
WT
R59W
GAPDH

Figure 2D

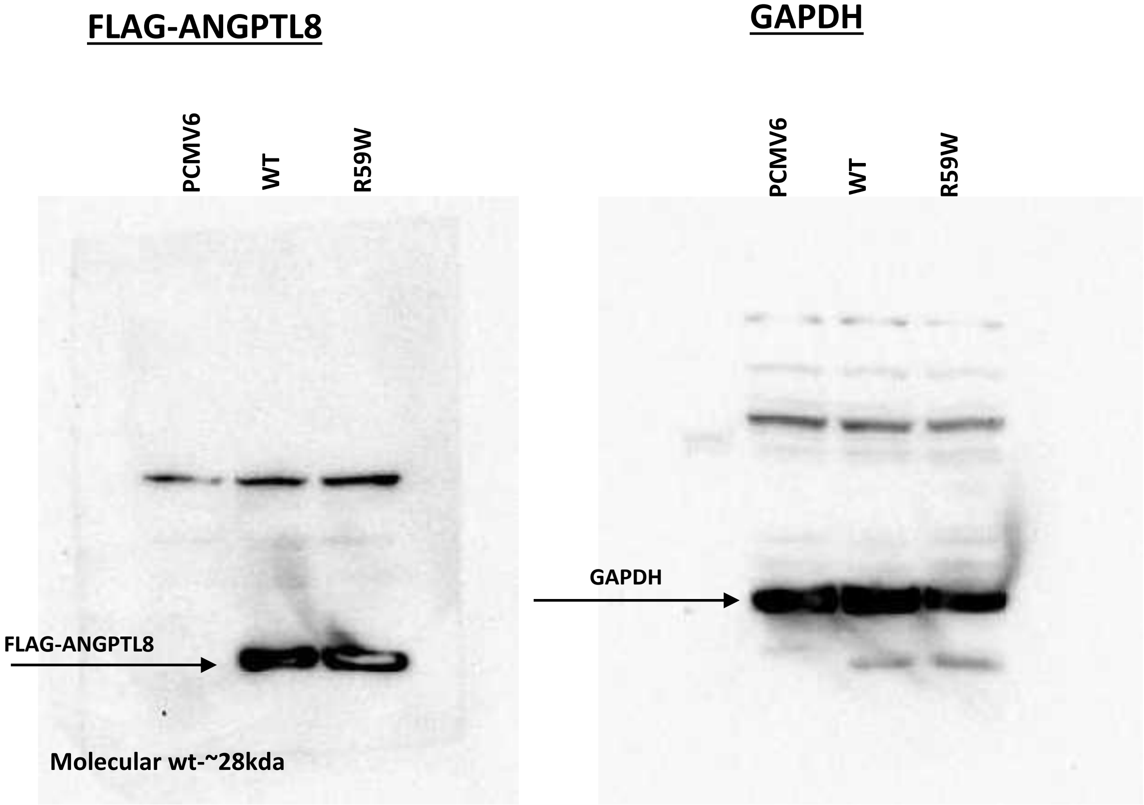
FLAG-ANGPTL8
PCMV6
WT
R59W
FLAG-ANGPTL8
Molecular wt-~28kda
GAPDH
PCMV6
WT
R59W
GAPDH

FIGURE 3A

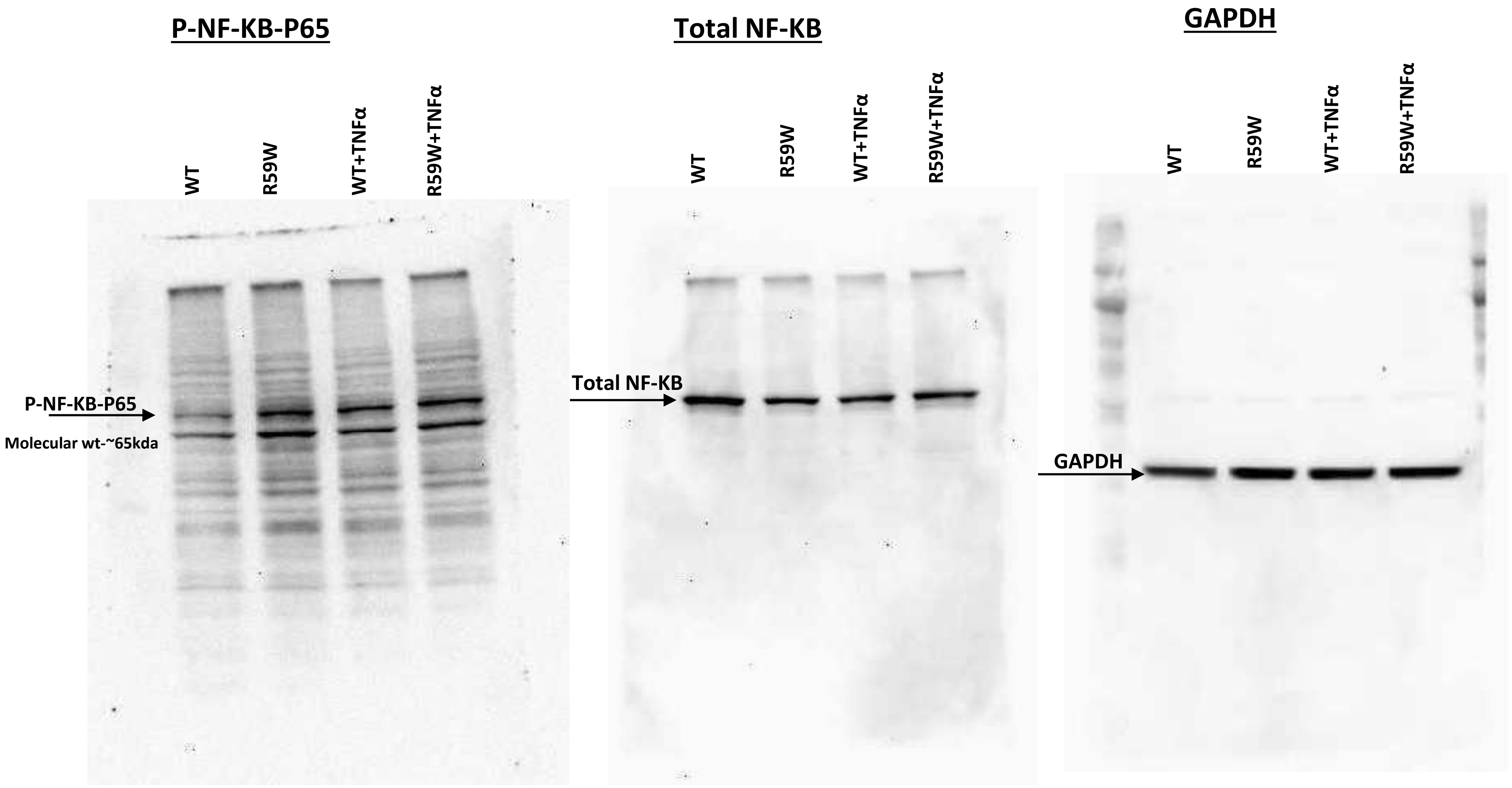
P-NF-KB-P65
WT
R59W
WT+TNFα
R59W+TNFα
P-NF-KB-P65
Molecular wt-~65kda
Total NF-KB
WT
R59W
WT+TNFα
R59W+TNFα
Total NF-KB
GAPDH
WT
R59W
WT+TNFα
R59W+TNFα
GAPDH

FIGURE 3B

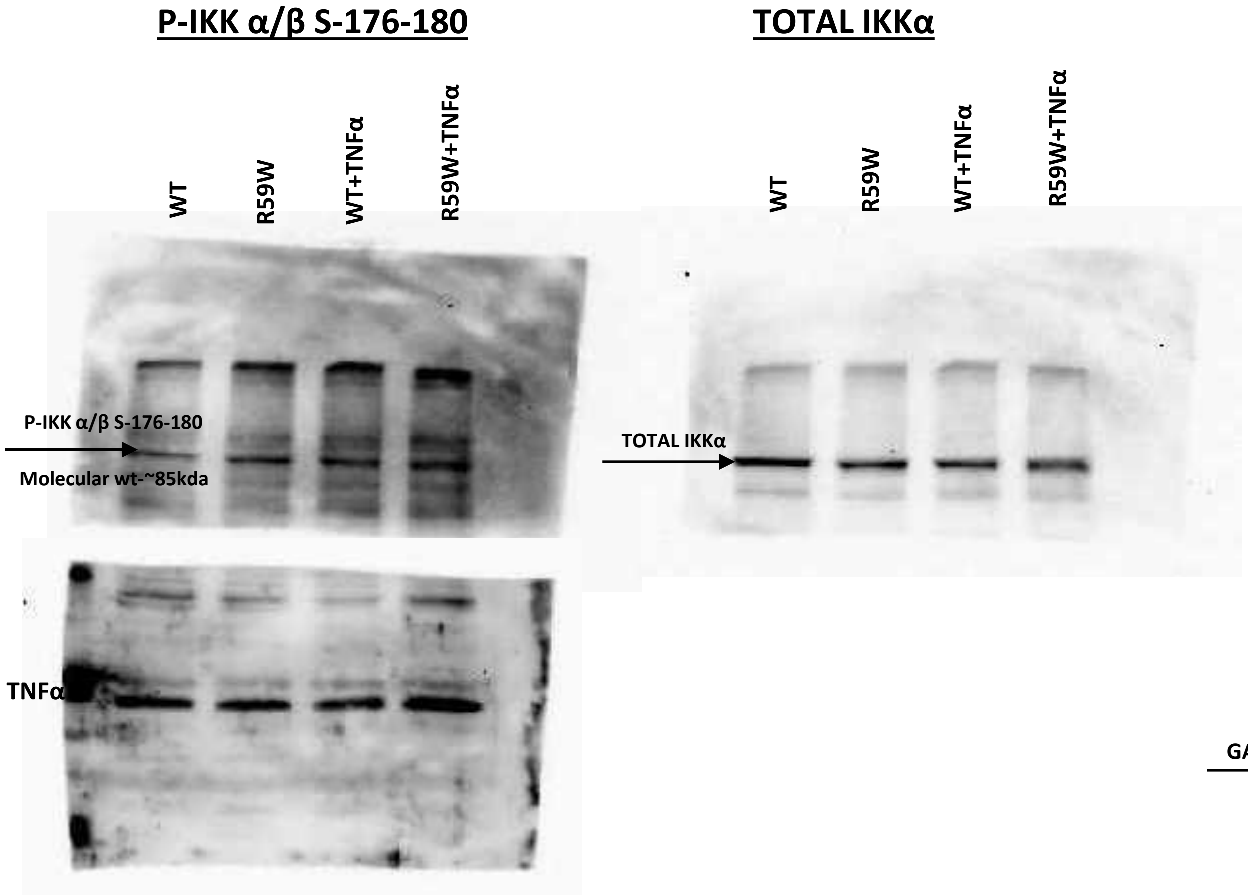

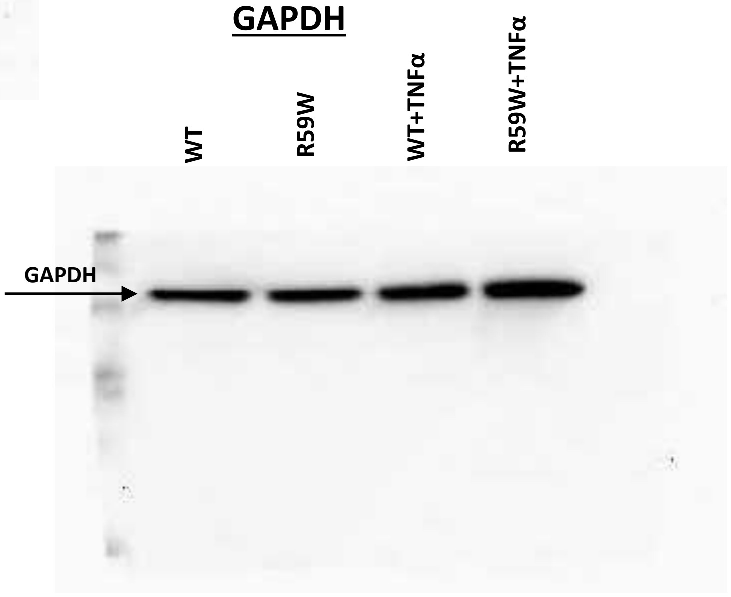

The blot was cut around 50kda, the upper half was blotted for P-IKK α/β , then for Total Ikkα . The bottom half was blotted for a TNFα(Fig-4A) and then for GAPDH

FIGURE 3C

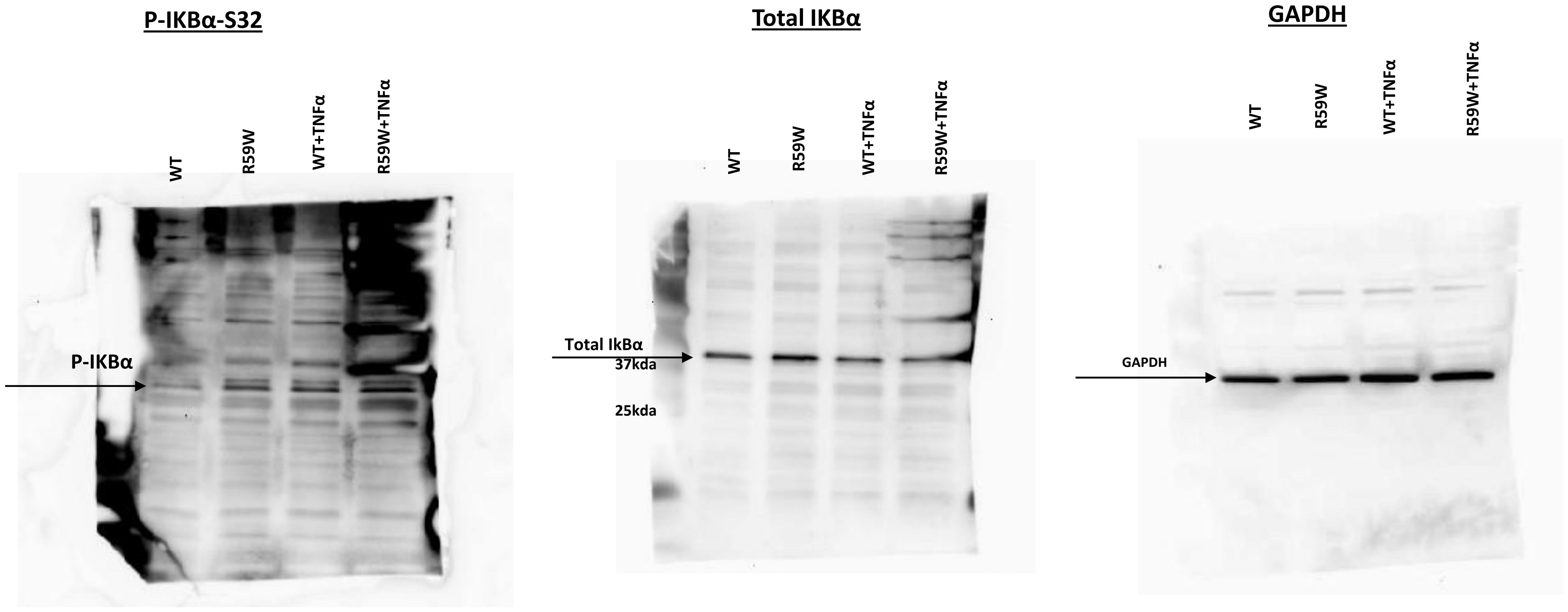
P-IKBα-S32
WT
R59W
WT+TNFα
R59W+TNFα
P-IKBα
Total IKBα
WT
R59W
WT+TNFα
R59W+TNFα
Total IkBα
37kda
25kda
GAPDH
WT
R59W
WT+TNFα
R59W+TNFα
GAPDH

Figure 4A

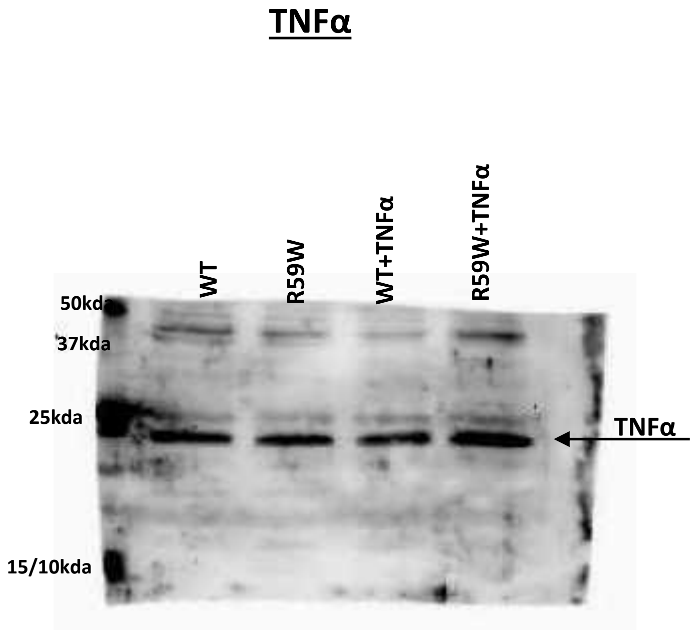

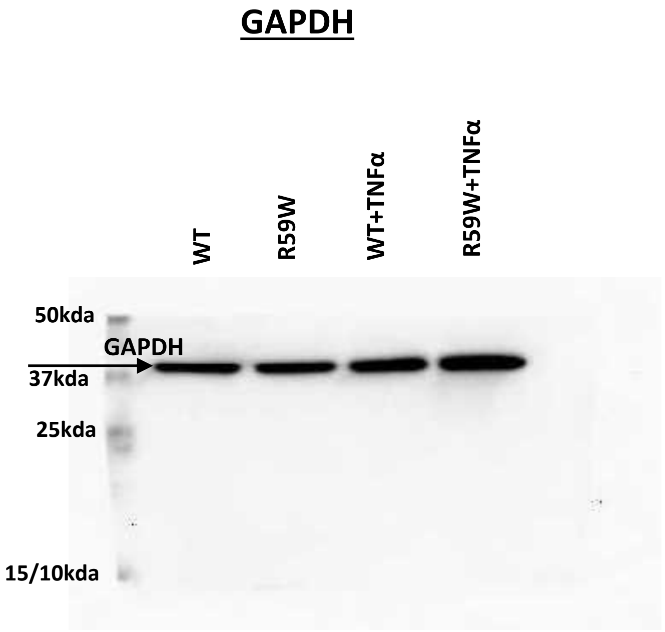

The blot was cut around 50kda, the upper half was blotted for P-IKK α/β(Fig3b) and the bottom half was blotted for a TNFα, then for GAPDH

Molecular wt-~24kda

FIGURE 4-A

IL-6

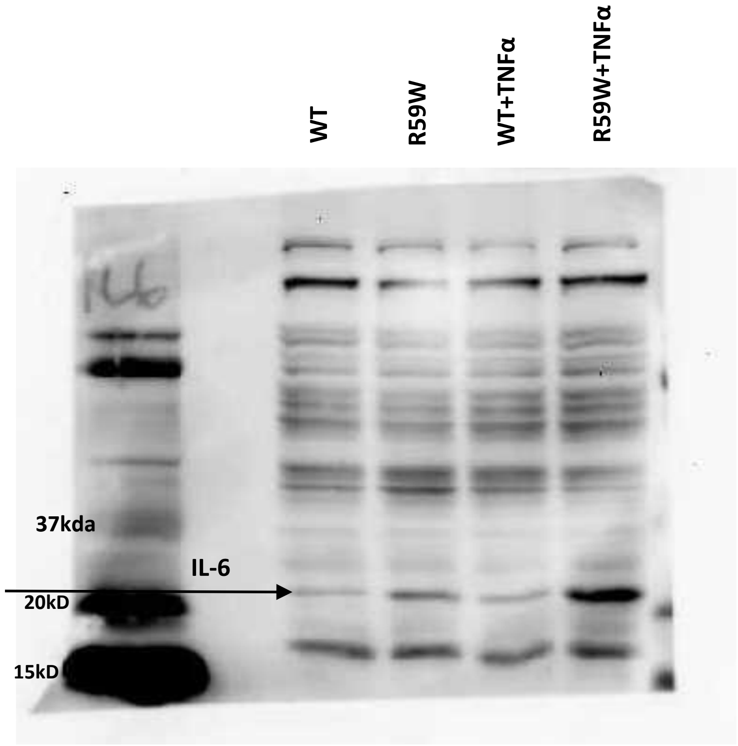

Molecular wt-~21kda

GAPDH

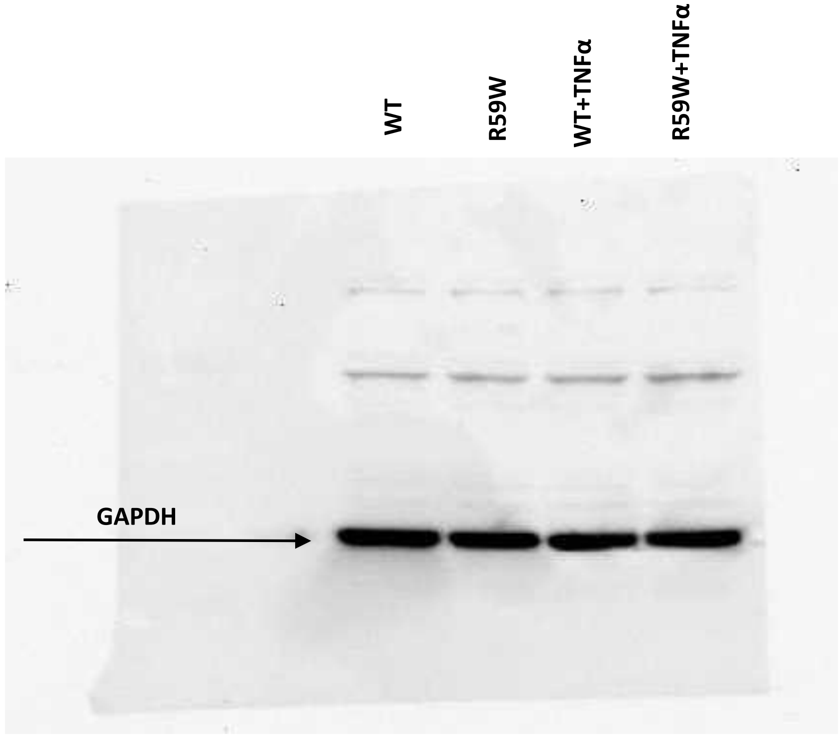

Supplement: Supplementary file 1 [file cells-12-02563-s001.zip › cells-2531520-supplementary.pdf]
